# A Thematic Analysis of Mental Health Student Perceptions of Working With Older Persons With Serious Mental Illness

**DOI:** 10.1093/geroni/igaa057.016

**Published:** 2020-12-16

**Authors:** Stephanie Santos, Michelle Zechner

**Affiliations:** 1 Rutgers University, Middlesex, New Jersey, United States; 2 Rutgers University, Piscataway, New Jersey, United States

## Abstract

Older people with serious mental illness (SMI) are a rapidly growing population that is expected to double in the US by 2030 (Pratt, Mueser, Bartels, & Wolfe, 2013). Negative attitudes towards older adults and people with SMI are reported for medical, nursing, and mental health students, but little research has examined mental health student attitudes about older adults living with SMI (Nochajski, et al., 2009; Smith & Cashwell, 2010). Education can improve attitudes towards vulnerable populations (Frailing & Slate, 2016). This retrospective qualitative study aimed to identify mental health profession student’s perceptions of working with older adults with SMI throughout a fifteen-week, online, mental health course, that educated students on the unique needs of older adults with SMI, available community services and resources, barriers to adequate services, and the effects of ageism. Undergraduate (n=13) and graduate (n=3) forum responses were gathered at three time points (Week 1, Week 5, and Week 15) to examine their attitudes about working with older adults with SMI. De-identified responses were aggregated. Conventional content analysis was used to identify themes. Two researchers manually coded aggregated information, and met to reach consensus regarding underlying themes. Content analysis suggested the following themes: negative feelings towards older adults, personal experiences with older adults, generational differences, cultural views of aging, attitudes about aging, and generational differences. Suggestions for future work with mental health students to reduce stigma and encourage them to work with older adults with SMI will be presented.

